# Neoantigens in cancer immunotherapy: focusing on alternative splicing

**DOI:** 10.3389/fimmu.2024.1437774

**Published:** 2024-07-11

**Authors:** Peng Huang, Feng Wen, Nuerye Tuerhong, Yang Yang, Qiu Li

**Affiliations:** ^1^ Division of Abdominal Tumor Multimodality Treatment, Cancer Center, West China Hospital, Sichuan University, Chengdu, Sichuan, China; ^2^ Department of Medical Oncology, Cancer Center, West China Hospital, Sichuan University, Chengdu, Sichuan, China; ^3^ Department of Radiation Oncology, Cancer Center, West China Hospital, Sichuan University, Chengdu, Sichuan, China

**Keywords:** alternative splicing, neoantigen, cancer, immunotherapy, CAR-T

## Abstract

Alternative splicing (AS) functions as a crucial program in transcriptional modulation, leading to proteomic diversity and functional alterations of proteins. These splicing actions induce various neoantigens that hold prognostic significance and contribute to various aspects of cancer progression, including immune responses against cancer. The advent of immunotherapy has remarkably revolutionized tumor therapy. In this regard, AS-derived neoantigens are potent targets for cancer vaccines and chimeric antigen receptor (CAR) T cell therapies. In this review, we outline that AS-derived neoantigens serve as promising immunotherapeutic targets and guide immunotherapy strategies. This evidence contributes to a deeper comprehension of the complexity of proteomic diversity and provides novel perspectives and techniques for precision medicine in immunotherapy. Moreover, we underscore the obstacles that are awaited to be addressed for this novel approach to become clinically applicable.

## 1 Introduction

Since the initial identification of eukaryotic “split” genes in 1977, featuring intervening sequences, it has become clear that these introns can be excised by the action of the spliceosome complex (1). Emerging studies have demonstrated that about 94% of human genes possess intronic regions during the pre-messenger RNA (mRNA) processing phase, and a significant portion of eukaryotic genes undergo alternative splicing in a manner dependent on both timing and spatial context (2). As a result, a single gene can give rise to alternatively spliced mRNAs that encode multiple proteins, each with unique functions, a strategy commonly utilized to maintain cellular homeostasis and to modulate cellular differentiation and development (3, 4). This adds a layer of complexity to mRNA, thereby increasing the variability in protein functions, which are commonly deployed to maintain cellular homeostasis and to modulate disease progression (1, 3, 4). The regulation and mechanics of the splicing process are crucial for comprehending the characteristic features of cancer, which hold immense potential for cancer therapy (5).

The accumulation of genetic mutations in cancers leads to the generation of tumor-specific antigens (TSAs) or neoantigens (6). These antigens can be presented by the tumor cells’ major histocompatibility (MHC) molecules (6). T cells recognize these specific peptide-MHC complexes, initiating an anti-cancer immune response in patients (7, 8). The introduction of immunotherapy has revolutionized the therapeutic strategies of numerous cancer types (9). Notable clinical outcomes have been achieved with cancer vaccines, adoptive T cell therapies, and antibodies that enhance T lymphocyte function (10). Furthermore, there is growing evidence that adaptive immunity plays a significant role in the enduring clinical benefits of traditional anticancer treatments like chemotherapy and radiotherapy (11, 12). Although cancer immunotherapy has led to remarkably durable response rates in some patients, a significant barrier to its wider use is the current shortage of known targetable neoantigens for many types of cancer (13, 14). Neoantigens provide a unique advantage due to their tumor-specific nature and absence in normal tissues, making them ideal targets for highly personalized tumor treatments. Expanding the population of neoantigen-specific T cells, due to their ability to circumvent central T cell tolerance, enhances tumor-specific immune responses (13, 14). Additionally, the potential of immunotherapy to boost neoantigen-specific T cell responses, providing lasting effects and post-treatment immunological memory, raises the possibility of long-term protection against disease recurrence (15, 16).

The focus on tumor cell neoantigens, predominantly from somatic mutations, frequently causes the neglect of potential neoepitopes produced through aberrant RNA splicing processes (8). Notably, mRNA splicing events have been demonstrated to contribute to tumor development, potentially expanding the target space for immunotherapy by producing neoepitopes (17, 18). The advent of next-generation sequencing technologies enables the widespread investigation of alternative splicing events in cancer and the economical identification of tumor-specific neoepitopes in individual patients (17, 18). Moreover, the creation of algorithms to predict the binding of epitopes to MHC molecules has facilitated the identification of potentially immunogenic neoepitopes, which may provide fresh perspectives on cancer immunotherapy and is expected to improve the clinical prognosis of cancer patients (19, 20). This review offers a comprehensive overview of the origins and biological roles of neoantigens produced by alternative splicing and the clinical uses of neoantigen-based immunotherapy approaches. Additionally, we explore the potential and challenges of applying neoantigen-based immunotherapies in clinical settings and prospect several possible solutions.

## 2 Concise view of the alternative splicing in cancer

The splicing program is facilitated through the combined efforts of various subunit complexes (1). At the core of the spliceosome presents five small ribosomal proteins (U1, U2, U5, and U4/U6) alongside a plethora of other proteins (2, 4). Cis-acting modulatory sequences, splicing factors, and additional RNA-binding proteins (RBPs) that identify and attach to splice sites are essential in forming and preserving patterns of alternative splicing (21, 22). These components are organized into specific precursor mRNAs (pre-mRNAs), facilitating the dynamic enzymatic process of splicing programs (21). This splicing reaction is initiated by the spliceosome identifying and attaching to critical cis-acting elements at exon-intron junctions (5′-splice and 3′-splice sites) in the pre-mRNA transcript, including branch points and polypyrimidine tracts within introns (23). This facilitates exon recognition and enables the spliceosome to carry out two subsequent transesterification reactions, leading to the excision of introns and the joining of exons (24, 25). In addition to the constitutive pre-mRNA splicing mechanism, the selective utilization of splice sites within a pre-mRNA can lead to the generation of mRNA and protein variants that differ both structurally and functionally (24, 25). This process, known as alternative splicing, contributes to biological complexity. Consequently, alternative splicing is pervasive, with over 90% of human pre-mRNAs undergoing this modification (1, 26). Various forms of alternative splicing have been characterized, such as the inclusion or exclusion of cassette exons, usage of alternative 5′ and 3′ splice sites, mutually exclusive exons, retention of introns, and the selection of alternative promoters and polyA sites (27, 28).

Growing evidence indicates that disruptions in the splicing process can play a significant role in the onset, development, and treatment resistance of cancer by altering the isoform expression of crucial proteins (28–30). For example, the alternative isoform of CD44, which is extensively studied across various cancer types, is linked to the epithelial-to-mesenchymal transition (31). The utilization of alternative 5′ splice sites in Bcl-x pre-mRNA results in the formation of the anti-apoptotic Bcl-x(L) and pro-apoptotic Bcl-x(S) protein isoforms (32, 33). Bcl-x(L) is transcriptionally upregulated in numerous cancers and is linked to chemoresistance, as well as to the RAS-induced expression of stemness regulators and the preservation of a cancer-initiating cell phenotype (34). Indeed, the simultaneous expression of mutant versions of SF3B1 and SRSF2 in hematopoietic progenitors has been demonstrated to induce cell death (35, 36). Conversely, the co-expression of mutant SRSF2 and the epigenetic modifier IDH2 leads to more significant splicing alterations than those caused by each mutation individually (37, 38). These mutations have synergistic impacts on both the epigenome and RNA splicing, which collectively enhance the progression of leukemia (37, 39).

It is commonly recognized that modifications in the splicing machinery can confer advantages to tumor cells, for instance, by producing atypical protein isoforms or modifying the proportions of standard cellular isoforms, albeit at the cost of diminished splicing efficiency or precision (40, 41). This fragile equilibrium is susceptible to disruption from further perturbations in splicing activity, such as mutations, the use of inhibitors, or heightened demand, leading to cytotoxic outcomes and thus highlighting splicing as a potential weak point in cancer cells (42, 43).

## 3 Alternative splicing as the source of tumor neoantigens

**Figure 1:**
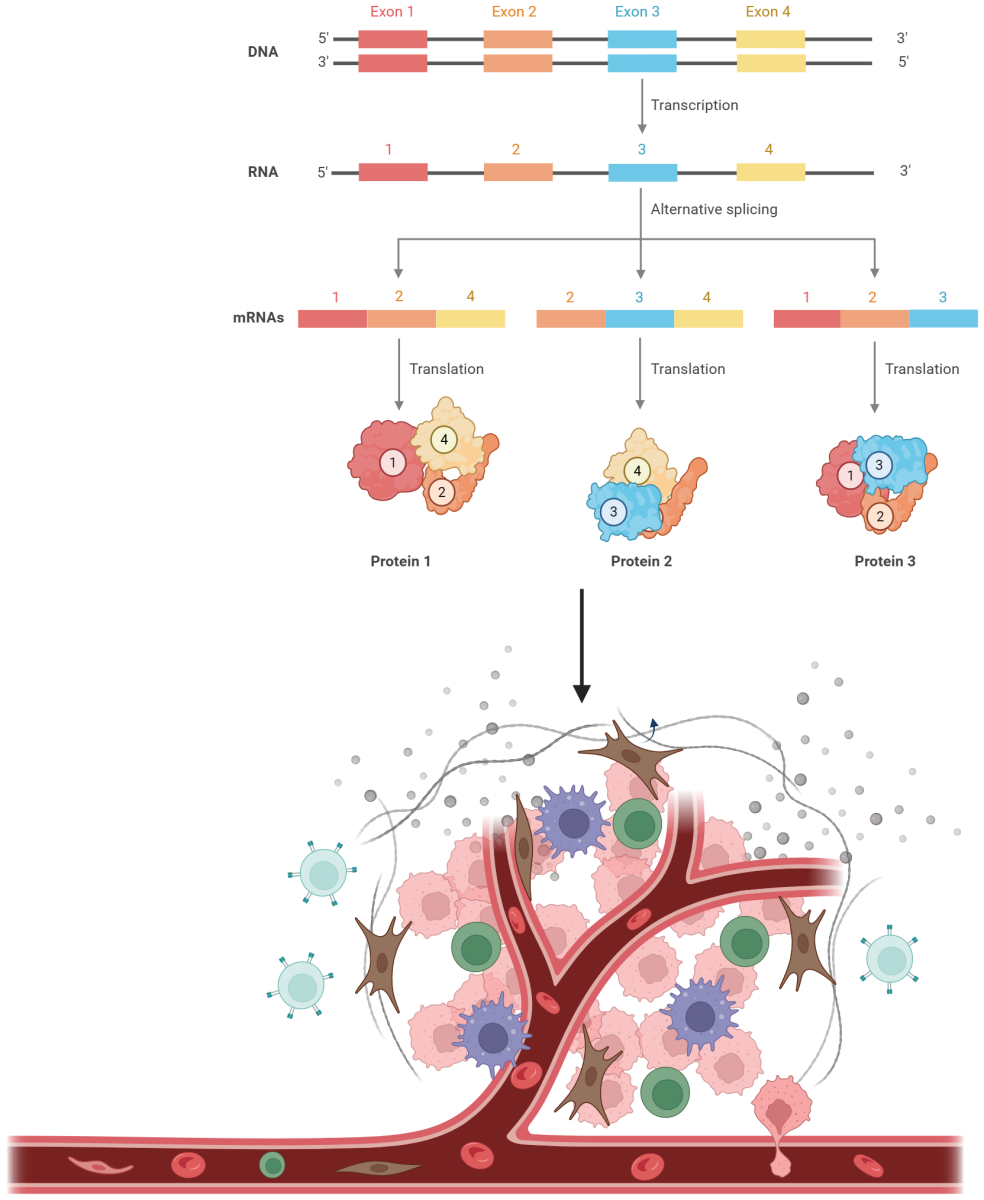
Alternative splicing as the source of neoantigens. A single pre-mRNA can be variably spliced into unique mature transcripts, resulting in the production of various proteins. Proteins derived from alternative splicing can be processed into small peptides. These peptides are then loaded onto major histocompatibility complex (MHC) class I. The peptide-MHC complexes can be recognized by T cells.

Recent studies have demonstrated that peptides originating from tumor-specific mRNA splicing events possess the capability to interact with MHC class I (MHC I) molecules, acting as neoepitopes (44, 45). A comprehensive The Cancer Genome Atlas (TCGA) study across various cancers suggested the involvement of alternative splicing programs in neoantigen generation by pinpointing cancer-specific exon-exon junctions and validating the presence of splicing-derived peptides through proteomics databases (46, 47). Furthermore, intron retention has been highlighted as a crucial mechanism for neoantigen prediction, as numerous intron-retaining neoantigens have been identified through transcriptome sequencing and mass spectrometry analyses (48). An examination of RNA sequencing data from 400 pediatric B-cell acute lymphoblastic leukemia (B-ALL) samples revealed a higher occurrence of the skipping event of the CD22 exon 5–6, a finding that was subsequently validated in 18 primary B-ALL samples (49). Additionally, this specific CD22 isoform, rather than the full-length version, could be targeted using a new, highly precise monoclonal antibody in the experimental mouse models (50). PTIR1 was specifically induced in human cancers through the alternative splicing of RIG-I (DDX58), and its induction correlated closely with unfavorable outcomes in cancer patients (51). In contrast to RIG-I, PTIR1 could bind to the C-terminus of UCHL5 and activate its ubiquitinating function, leading to the inhibition of immunoproteasome activity and the restriction of neoantigen processing and presentation, thereby impeding T cell recognition and immune attack on cancer (51). In medulloblastoma, a cancer known for its low mutation rate, neoantigens were predominantly formed through abnormal splice junctions connecting two non-exonic sequences (52). The manipulation of splicing programs through pharmacological interventions also impacts the production of neoepitopes (53). The application of splicing modulators like indisulam in both laboratory settings and living organisms across different cancer types has led to the creation of splicing-induced neoantigens that positively influence tumor immunogenicity (53). This strategy indicates the potential for targeting alternative splicing in human cancers to augment existing immune responses (**Figure 1**).

## 4 AS-derived neoantigens in tumor immunotherapy

Neoantigens, which are unique to cancer and not present in normal tissues, serve as prime targets for immune surveillance against tumors by T cells (44). However, the effectiveness of current immunotherapies focusing on neoantigen-specific T-cell responses is hindered by limited neoantigen expression levels, a lack of common antigen targets among patients, and the diverse genetic makeup within tumors (54). In a recent preprint manuscript, Kwok and colleagues analyzed RNA sequencing data from TCGA and their repository of brain cancer samples, they pinpointed shared junction reads across patients and multiple sites within the same tumors (44). Employing computational models for antigen processing, the researchers pinpointed HLA-A*02:01-restricted peptides derived from neojunctions for further scrutiny. Mass spectrometry analysis of HLA-I ligands validated the endogenous processing and presentation of these antigens (44). They engineered CD8+ T cells with modified T cell receptors (TCRs) aiming at these mutations and effectively eliminated tumor cells specific to the neoantigens. Moreover, the alterations in splicing factors specific to certain cancer subtypes that could potentially regulate the expression of neojunction-derived antigens were reported (44, 54). These findings offer an extensive overview of preserved neoantigens resulting from AS events, capable of eliciting robust T-cell reactions. *In vivo* investigations are essential to assess tumor infiltration and regulation by T cells responsive to these AS-derived antigens. The ability to induce the expression of neoantigens in mice would facilitate the investigation of innate neoantigen-specific T-cell responses in the context of cancer. However, this has proven challenging to achieve due to the unintended leakage of antigen expression in the thymus, leading to the development of central T cell tolerance. Recently, Damo et al. introduced the concept of inversion-induced joined neoantigen (NINJA), utilizing RNA splicing, and a sophisticated regulatory system to prevent unintended expression and ensure precise control over neoantigen presentation (55). By the NINJA tool, they successfully established tumor cell lines with controllable neoantigen expression, and genetic modulatory programs in NINJA mice inhibited both central and peripheral tolerance processes, leading to robust activation of CD8+ T cells upon neoantigen stimulation (55). Moreover, Masahiko et al. identified a small splicing modulator-RECTAS that acted specifically to rectify abnormal splicing by enhancing the function of Serine/arginine-rich splicing factor 6 (SRSF6) (56). In the colorectal cancer mouse models, RECTAS treatment triggered AS programs that induced six potential neoantigens, which could exacerbate T cell responses capable of eliminating cancer cells in laboratory settings and lead to tumor growth suppression *in vivo* (57).

The expression of PD-L1 by tumors suppresses the anti-tumor immune response, allowing tumors to escape immune surveillance (58). The targeting of PD-1 and its ligand PD-L1 has demonstrated remarkable clinical efficacy in clinical studies (59, 60). PD-1 undergoes alternative splicing to generate isoforms that exist as either transmembrane signaling receptors responsible for inducing T cell death through interaction with the ligand PD-L1 or as an alternatively spliced soluble variant devoid of the transmembrane domain (61). And skipping exon 3 could generate the soluble PD-1, which was naturally produced in peripheral blood mononuclear cells and T cells, preventing cancer cells from suppressing T cell activity, which was regulated by SRSF1 and SR protein kinase 1 (SRPK1) (62). Notably, inhibiting SRPK1 altered the splicing program to produce the antagonistic isoform of PD-1, boosting T-cell destruction of cancer cells, and implying the potential application of small molecules targeting SRPK1 as innovative pharmacological immunotherapies (62). Kathleen et al. presented a secreted splice variant of PD-L1 (secPD-L1) that hindered the activation of T cells in laboratory settings (63). This variant was present in PD-L1-positive tumor cells as well as in PD-L1-positive normal tissues. SecPD-L1 necessitated higher concentrations in laboratory conditions, indicating its potential for heightened activity in the tumor microenvironment, which provided a novel mechanism for mediating immunosuppression within the tumor microenvironment in a paracrine manner (63). Moreover, the AS program plays a significant role in the resistance to immune checkpoint inhibitors. For instance, secPD-L1 could compete with PD-L1 antibodies for binding, leading to resistance to PD-L1 antibody therapy. The chemical activation of SRSFs induced by splicing modulator RECTAS produced several neoantigens that enhanced the effectiveness of PD-1 blockades (57), suggesting the investigations of splicing modifications in tumors could provide indicators for utilizing immune-checkpoint inhibitors like anti-PD-1 or anti-CTLA-4 antibodies.

The discovery of neoantigens derived from splicing could also serve as a valuable predictive marker for how patients respond to immune checkpoint inhibitor (ICI) therapy (64, 65). Studies have indicated a link between tumor mutational burden (TMB) and the presence of neoantigens on MHC molecules (66). While TMB has correlated with responses to ICI therapy in various cancers, it’s not the sole determinant (67–69). Some high-TMB patients responded poorly, while some low-TMB patients responded well to ICIs (70, 71). A load of splicing-derived neoepitopes could potentially be a clinical biomarker for ICI response (72). Challenges remain in identifying tumor-specific splicing events and validating the immunogenicity and specificity of these neoantigens (64, 72).

**Figure 2:**
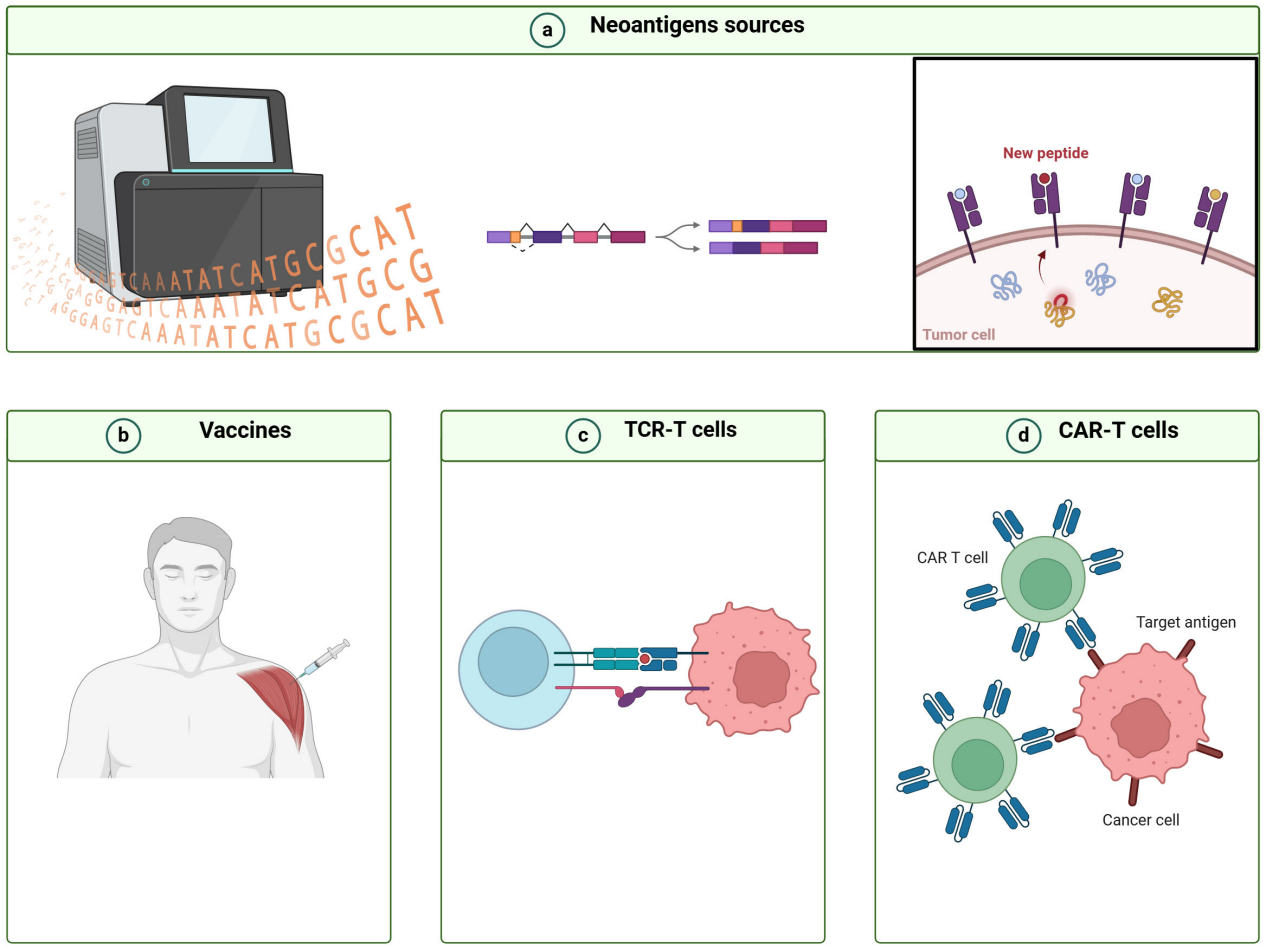
Workflow for designing immunotherapy strategies based on cancer neoantigens derived from alternative splicing. **(A)** Bioinformatics tools are utilized to detect alternative splicing (AS) events, analyze peptides derived from AS, and forecast their immunogenicity. **(B)** Novel cancer vaccines, **(C)** T cell receptor-engineered T (TCR-T) cells, and **(D)** chimeric antigen receptor T (CAR-T) cells are designed utilizing immunogenic AS-derived neoantigens.

The emergence of neoantigens through modified splicing processes, such as intron retention, intron polyadenylation, and fusion transcript formation, offers significant prospects for the advancement of cancer vaccines and therapies involving chimeric antigen receptor (CAR) T cells and T cell receptor-engineered T (TCR-T) cells (73, 74) (**Figure 2**). For instance, U2AF1^Q157R^ neoantigens were derived from the mutations in the spliceosome (75). The lentiviral transfer of U2AF1^Q157R^ neoantigen-specific TCRs imparted epitope specificity to CD8+ T cells from other donors. These U2AF1^Q157R^ neoantigen-specific TCR-T cells effectively targeted and eliminated U2AF1^Q157R^ -expressing malignant myeloid cell lines *in vitro*, while sparing non-malignant hematopoietic stem/progenitor cells (75). Furthermore, in a cell line-derived xenograft murine model, U2AF1^Q157R^ neoantigen-specific TCR-T cells also demonstrated the ability to kill neoplastic myeloid cells harboring the U2AF1^Q157R^ mutation effectively (75). These findings suggest that U2AF1Q157R neoantigens hold promise as targets for precision medicine approaches, including TCR-T cell therapy, in individuals with myeloid neoplasms, harboring these mutations.

Fibronectin (FN) produced from the alternative splicing of FN1 serves as a valuable source of antigens. Extra domain A (EDA) was an AS-related neoantigen generated by FN, which was overexpressed in multiple types of human cancers. Based on this specific FN isoform, EDA CAR-T cells were established for killing EDA-expressing cancer cells and achieved remarkable antitumor activity and no toxicity in experimental mouse models (76). Elisabeth et al. developed a vaccine targeted EDB, which could significantly reduce tumor size in vaccinated mice, providing promising candidates for the development of therapeutic vaccines aimed at targeting solid tumors (77). CD44v6 is a spliced isoform of CD44, induced by the inclusion of the sixth variant exon. CD44v6 is implicated in diverse biological functions such as cell proliferation, and angiogenesis. Recent studies have revealed that CD44v6 functions as a potent for CAR-T therapy. CD44v6-targeted CAR-T cells expressing a suicide gene had effectively eliminated acute myeloid leukemia (AML) *in vivo* (78). And it might specifically target AML patients harboring FMS-like tyrosine kinase 3 (FLT3) or DNA methyltransferase 3A (DNMT3A) mutations (79), which holds immense potential for clinical application but awaits further in-depth investigations.

## 5 Challenges and perspectives for AS-based neoantigens in cancer immunotherapy

Modulating splicing to alter the production of neoantigens at the RNA level offers several advantages. Splicing modulation has the potential to generate a large number of neoantigens, in contrast to the limited canonical neoantigens resulting from somatic mutations (53). While canonical neoantigens are often similar to their wild-type counterparts, splicing-derived peptides are frequently novel to the immune system, leading to significantly enhanced immune responses, as demonstrated in the study where 28–43% of predicted splicing-derived neoantigens were found to be immunogenic compared to an estimated 6% for mutation-derived neoantigens (53, 80).

Recent advancements in technology have enabled the identification of tumor-specific mRNA splicing-derived neoantigens. These neoantigens have the potential to introduce a novel category of targets for cancer immunotherapy. However, there are several challenges that persist in the development and implementation of immunotherapies aimed at targeting these mRNA splicing-derived neoantigens. (1) How to identify tumor-specific splicing events. Accurately identifying mRNA splicing events specific to tumors will be crucial for the effectiveness of immunotherapies targeting neoantigens (81). The advancement of RNA sequencing (RNA-seq) technologies provided available tools for screening AS events, However, the technique lacks the ability to detect splicing effects at the subclone level (82, 83). Consequently, therapies targeting events present in only a fraction of the tumor may prove ineffective. The emergence of single-cell RNA-seq (scRNA-seq) holds promise for detecting splicing events across all tumor cells (84, 85). However, integrating alternative splicing analysis with scRNA-seq remains technically challenging, as scRNA-seq is constrained by a limited starting material, potentially limiting the analysis to highly abundant transcripts (86). Addressing this issue may be possible through the development of machine learning algorithms like the recently introduced DARTS, which has enhanced the characterization of splicing variations in transcripts with minimal coverage (87). (2) How to improve specificity. AS program exhibits variability due to the dynamic action of the spliceosome, leading to stochastic fluctuations, which can target the splicing isoform in healthy tissues (88, 89). Therefore, it is essential to implement adequate controls during the process of identifying tumor-specific peptides derived from splicing or significantly enriching the targeted splicing-derived peptides within tumors (90, 91). (3) Ensuring effectiveness and safety in the clinical setting. Since splicing inhibition could have the risk of causing side effects such as inflammatory responses and developmental defects, safety needs to be carefully monitored in additional species than mice before clinical trials are initiated (92, 93). Furthermore, emerging evidence has revealed several immune escape mechanisms that interfered with the immunological presentation of AS-derived neoantigens (66, 94). It remains to be established whether memory T-cells are generated in response to these AS-derived neoantigens and whether any immune response persists after the cessation of the splicing inhibitors (95, 96).

## 6 Conclusion

Increasing evidence suggests that alternative mRNA splicing-derived neoepitopes hold immense potential as immunotherapeutic targets that may significantly improve the prognosis of cancer patients. Despite significant progress in broadening the immunotherapy target landscape using RNA-seq technologies, extensive work is still required. Therefore, there is a demand for new technologies that enable rapid, robust, and precise identification of tumor-specific immunogenic epitopes to accurately evaluate their therapeutic potential. Furthermore, incorporating AS-derived neoepitopes as potential targets for cell-based and/or vaccination-based immunotherapeutic anticancer strategies may extend the benefits of such treatments to a broader range of patients.

## Author contributions

PH: Conceptualization, Data curation, Investigation, Methodology, Software, Writing – original draft, Writing – review & editing. FW: Conceptualization, Data curation, Funding acquisition, Investigation, Methodology, Writing – review & editing. NT: Conceptualization, Data curation, Investigation, Methodology, Writing – review & editing. YY: Data curation, Formal analysis, Investigation, Writing – review & editing. QL: Funding acquisition, Project administration, Resources, Supervision, Validation, Writing – review & editing.

## References

[1] Marasco LE Kornblihtt AR. The physiology of alternative splicing. Nat Rev Mol Cell Biol. (2023) 24:242–54. doi: 10.1038/s41580-022-00545-z 36229538

[2] Baralle FE Giudice J. Alternative splicing as a regulator of development and tissue identity. Nat Rev Mol Cell Biol. (2017) 18:437–51. doi: 10.1038/nrm.2017.27 PMC6839889 28488700

[3] Bhadra M Howell P Dutta S Heintz C Mair WB. Alternative splicing in aging and longevity. Hum Genet. (2020) 139:357–69. doi: 10.1007/s00439-019-02094-6 PMC8176884 31834493

[4] Lee Y Rio DC. Mechanisms and regulation of alternative pre-mRNA splicing. Annu Rev Biochem. (2015) 84:291–323. doi: 10.1146/annurev-biochem-060614-034316 25784052 PMC4526142

[5] Bonnal SC López-Oreja I Valcárcel J. Roles and mechanisms of alternative splicing in cancer - implications for care. Nat Rev Clin Oncol. (2020) 17:457–74. doi: 10.1038/s41571-020-0350-x 32303702

[6] Katsikis PD Ishii KJ Schliehe C. Challenges in developing personalized neoantigen cancer vaccines. Nat Rev Immunol. (2024) 24:213–27. doi: 10.1038/s41577-023-00937-y PMC12001822 37783860

[7] Mariuzza RA Wu D Pierce BG. Structural basis for T cell recognition of cancer neoantigens and implications for predicting neoepitope immunogenicity. Front Immunol. (2023) 14:1303304. doi: 10.3389/fimmu.2023.1303304 38045695 PMC10693334

[8] Xu R Du S Zhu J Meng F Liu B. Neoantigen-targeted TCR-T cell therapy for solid tumors: How far from clinical application. Cancer Lett. (2022) 546:215840. doi: 10.1016/j.canlet.2022.215840 35921969

[9] Riley RS June CH Langer R Mitchell MJ. Delivery technologies for cancer immunotherapy. Nat Rev Drug Discovery. (2019) 18:175–96. doi: 10.1038/s41573-018-0006-z PMC6410566 30622344

[10] Licata L Dieci MV De Angelis C Marchiò C Miglietta F Cortesi L. Navigating practical challenges in immunotherapy for metastatic triple negative breast cancer. Cancer Treat Rev. (2024) 128:102762. doi: 10.1016/j.ctrv.2024.102762 38776613

[11] Pointer KB Pitroda SP Weichselbaum RR. Radiotherapy and immunotherapy: open questions and future strategies. Trends Cancer. (2022) 8:9–20. doi: 10.1016/j.trecan.2021.10.003 34740553

[12] Yu WD Sun G Li J Xu J Wang X. Mechanisms and therapeutic potentials of cancer immunotherapy in combination with radiotherapy and/or chemotherapy. Cancer Lett. (2019) 452:66–70. doi: 10.1016/j.canlet.2019.02.048 30902563

[13] Pang Z Lu MM Zhang Y Gao Y Bai JJ Gu JY. Neoantigen-targeted TCR-engineered T cell immunotherapy: current advances and challenges. biomark Res. (2023) 11:104. doi: 10.1186/s40364-023-00534-0 38037114 PMC10690996

[14] Zhang Z Lu M Qin Y Gao W Tao L Su W. Neoantigen: A new breakthrough in tumor immunotherapy. Front Immunol. (2021) 12:672356. doi: 10.3389/fimmu.2021.672356 33936118 PMC8085349

[15] Lang F Schrörs B Löwer M Türeci Ö Sahin U. Identification of neoantigens for individualized therapeutic cancer vaccines. Nat Rev Drug Discovery. (2022) 21:261–82. doi: 10.1038/s41573-021-00387-y PMC7612664 35105974

[16] Redwood AJ Dick IM Creaney J Robinson BWS. What’s next in cancer immunotherapy? - The promise and challenges of neoantigen vaccination. Oncoimmunology. (2022) 11:2038403. doi: 10.1080/2162402x.2022.2038403 35186441 PMC8855878

[17] Boesch M Baty F Rothschild SI Tamm M Joerger M Früh M. Tumour neoantigen mimicry by microbial species in cancer immunotherapy. Br J Cancer. (2021) 125:313–23. doi: 10.1038/s41416-021-01365-2 PMC8329167 33824481

[18] Schumacher TN Schreiber RD. Neoantigens in cancer immunotherapy. Science. (2015) 348:69–74. doi: 10.1126/science.aaa4971 25838375

[19] Aparicio B Theunissen P Hervas-Stubbs S Fortes P Sarobe P. Relevance of mutation-derived neoantigens and non-classical antigens for anticancer therapies. Hum Vaccin Immunother. (2024) 20:2303799. doi: 10.1080/21645515.2024.2303799 38346926 PMC10863374

[20] Zou J Zhang Y Pan Y Mao Z Chen X. Advancing nanotechnology for neoantigen-based cancer theranostics. Chem Soc Rev. (2024) 53:3224–52. doi: 10.1039/d3cs00162h 38379286

[21] Wang S DeLeon C Sun W Quake SR Roth BL Südhof TC. Alternative splicing of latrophilin-3 controls synapse formation. Nature. (2024) 626:128–35. doi: 10.1038/s41586-023-06913-9 PMC10830413 38233523

[22] Zheng Z Zeng X Zhu Y Leng M Zhang Z Wang Q. CircPPAP2B controls metastasis of clear cell renal cell carcinoma via HNRNPC-dependent alternative splicing and targeting the miR-182-5p/CYP1B1 axis. Mol Cancer. (2024) 23:4. doi: 10.1186/s12943-023-01912-w 38184608 PMC10770969

[23] Cui D Wang Z Dang Q Wang J Qin J Song J. Spliceosome component Usp39 contributes to hepatic lipid homeostasis through the regulation of autophagy. Nat Commun. (2023) 14:7032. doi: 10.1038/s41467-023-42461-6 37923718 PMC10624899

[24] Bowling EA Wang JH Gong F Wu W Neill NJ Kim IS. Spliceosome-targeted therapies trigger an antiviral immune response in triple-negative breast cancer. Cell. (2021) 184:384–403.e321. doi: 10.1016/j.cell.2020.12.031 33450205 PMC8635244

[25] Levesque L Salazar N Roy SW. Distinct minor splicing patterns across cancers. Genes (Basel). (2022) 13(2). doi: 10.3390/genes13020387 PMC8871696 35205431

[26] Ule J Blencowe BJ. Alternative splicing regulatory networks: functions, mechanisms, and evolution. Mol Cell. (2019) 76:329–45. doi: 10.1016/j.molcel.2019.09.017 31626751

[27] Hewezi T. Phytopathogens reprogram host alternative mRNA splicing. Annu Rev Phytopathol. (2024). doi: 10.1146/annurev-phyto-121423-041908 38691872

[28] Szelest M Giannopoulos K. Biological relevance of alternative splicing in hematologic Malignancies. Mol Med. (2024) 30:62. doi: 10.1186/s10020-024-00839-2 38760666 PMC11100220

[29] Kansara S Sawant P Kaur T Garg M Pandey AK. LncRNA-mediated orchestrations of alternative splicing in the landscape of breast cancer. Biochim Biophys Acta Gene Regul Mech. (2024) 1867:195017. doi: 10.1016/j.bbagrm.2024.195017 38341138

[30] Li J Huang G. Insulin receptor alternative splicing in breast and prostate cancer. Cancer Cell Int. (2024) 24:62. doi: 10.1186/s12935-024-03252-1 38331804 PMC10851471

[31] Hassn Mesrati M Syafruddin SE Mohtar MA Syahir A. CD44: A multifunctional mediator of cancer progression. Biomolecules. (2021) 11(12). doi: 10.3390/biom11121850 PMC8699317 34944493

[32] Chen W Li J. Alternative splicing of BCL-X and implications for treating hematological Malignancies. Oncol Lett. (2021) 22:670. doi: 10.3892/ol.2021.12931 34345295 PMC8323006

[33] Dou Z Zhao D Chen X Xu C Jin X Zhang X. Aberrant Bcl-x splicing in cancer: from molecular mechanism to therapeutic modulation. J Exp Clin Cancer Res. (2021) 40:194. doi: 10.1186/s13046-021-02001-w 34118966 PMC8196531

[34] Le Sénéchal R Keruzoré M Quillévéré A Loaëc N Dinh VT Reznichenko O. Alternative splicing of BCL-x is controlled by RBM25 binding to a G-quadruplex in BCL-x pre-mRNA. Nucleic Acids Res. (2023) 51:11239–57. doi: 10.1093/nar/gkad772 PMC10639069 37811881

[35] Cieśla M Ngoc PCT Muthukumar S Todisco G Madej M Fritz H. m(6)A-driven SF3B1 translation control steers splicing to direct genome integrity and leukemogenesis. Mol Cell. (2023) 83:1165–1179.e1111. doi: 10.1016/j.molcel.2023.02.024 36944332

[36] Zhou Z Gong Q Wang Y Li M Wang L Ding H. The biological function and clinical significance of SF3B1 mutations in cancer. biomark Res. (2020) 8:38. doi: 10.1186/s40364-020-00220-5 32905346 PMC7469106

[37] Jia W Guo X Wei Y Liu J Can C Wang R. Clinical and prognostic profile of SRSF2 and related spliceosome mutations in patients with acute myeloid leukemia. Mol Biol Rep. (2023) 50:6601–10. doi: 10.1007/s11033-023-08597-w 37344641

[38] Zorzan E Hanssens K Giantin M Dacasto M Dubreuil P. Mutational hotspot of TET2, IDH1, IDH2, SRSF2, SF3B1, KRAS, and NRAS from human systemic mastocytosis are not conserved in canine mast cell tumors. PloS One. (2015) 10:e0142450. doi: 10.1371/journal.pone.0142450 26562302 PMC4643045

[39] Eckardt JN Stasik S Röllig C Sauer T Scholl S Hochhaus A. Alterations of cohesin complex genes in acute myeloid leukemia: differential co-mutations, clinical presentation and impact on outcome. Blood Cancer J. (2023) 13:18. doi: 10.1038/s41408-023-00790-1 36693840 PMC9873811

[40] Del Giudice M Foster JG Peirone S Rissone A Caizzi L Gaudino F. FOXA1 regulates alternative splicing in prostate cancer. Cell Rep. (2022) 40:111404. doi: 10.1016/j.celrep.2022.111404 36170835 PMC9532847

[41] Wang X Hua J Li J Zhang J Dzakah EE Cao G. Mechanisms of non-coding RNA-modulated alternative splicing in cancer. RNA Biol. (2022) 19:541–7. doi: 10.1080/15476286.2022.2062846 PMC9037454 35427215

[42] Ruta V Naro C Pieraccioli M Leccese A Archibugi L Cesari E. An alternative splicing signature defines the basal-like phenotype and predicts worse clinical outcome in pancreatic cancer. Cell Rep Med. (2024) 5:101411. doi: 10.1016/j.xcrm.2024.101411 38325381 PMC10897606

[43] Xu T Li X Zhao W Wang X Jin L Feng Z. SF3B3-regulated mTOR alternative splicing promotes colorectal cancer progression and metastasis. J Exp Clin Cancer Res. (2024) 43:126. doi: 10.1186/s13046-024-03053-4 38671459 PMC11047005

[44] Puig-Saus C Sennino B Peng S Olcese C Pang N Patterson E. Neoantigen-targeted CD8(+) T cell responses with PD-1 blockade therapy. Nature. (2023) 615:697–704. doi: 10.1038/s41586-023-05787-1 36890230 PMC10441586

[45] Rojas LA Sethna Z Soares KC Olcese C Pang N Patterson E. Personalized RNA neoantigen vaccines stimulate T cells in pancreatic cancer. Nature. (2023) 618:144–50. doi: 10.1038/s41586-023-06063-y PMC10171177 37165196

[46] Liu S Matsuzaki J Wei L Tsuji T Battaglia S Hu Q. Efficient identification of neoantigen-specific T-cell responses in advanced human ovarian cancer. J Immunother Cancer. (2019) 7:156. doi: 10.1186/s40425-019-0629-6 31221207 PMC6587259

[47] Pulakuntla S Syed K Reddy VD. Analysis of somatic mutations in the TCGA-LIHC whole exome sequence to identify the neoantigen for immunotherapy in hepatocellular carcinoma. Curr Issues Mol Biol. (2023) 46:106–20. doi: 10.3390/cimb46010009 PMC10813969 38248311

[48] Lao Y Wang Y Yang J Liu T Ma Y Luo Y. Characterization of genomic alterations and neoantigens and analysis of immune infiltration identified therapeutic and prognostic biomarkers in adenocarcinoma at the gastroesophageal junction. Front Oncol. (2022) 12:941868. doi: 10.3389/fonc.2022.941868 36439494 PMC9691957

[49] Aslar Oner D Akin DF Sipahi K Mumcuoglu M Ezer U Kürekci AE. Screening of variations in CD22 gene in children with B-precursor acute lymphoblastic leukemia. Genet Test Mol Biomarkers. (2016) 20:552–5. doi: 10.1089/gtmb.2016.0006 27486888

[50] Zheng S Naqvi AS Bolton-Gillespie E Asnani M Hayer K Barash Y. Pipeline for discovering neoepitopes generated by alternative splicing in B-ALL. Blood. (2019) 134:1342. doi: 10.1182/blood-2019-131277

[51] Song J Liu Y Yin Y Wang H Zhang X Li Y. PTIR1 acts as an isoform of DDX58 and promotes tumor immune resistance through activation of UCHL5. Cell Rep. (2023) 42:113388. doi: 10.1016/j.celrep.2023.113388 37934668

[52] Blaeschke F Paul MC Schuhmann MU Rabsteyn A Schroeder C Casadei N. Low mutational load in pediatric medulloblastoma still translates into neoantigens as targets for specific T-cell immunotherapy. Cytotherapy. (2019) 21:973–86. doi: 10.1016/j.jcyt.2019.06.009 31351799

[53] Lu SX De Neef E Thomas JD Sabio E Rousseau B Gigoux M. Pharmacologic modulation of RNA splicing enhances anti-tumor immunity. Cell. (2021) 184:4032–4047.e4031. doi: 10.1016/j.cell.2021.05.038 34171309 PMC8684350

[54] Brown M Vabret N. Alternative RNA splicing generates shared clonal neoantigens across different types of cancer. Nat Rev Immunol. (2024) 24:160. doi: 10.1038/s41577-023-00986-3 38216760

[55] Damo M Fitzgerald B Lu Y Nader M William I Cheung JF. Inducible *de novo* expression of neoantigens in tumor cells and mice. Nat Biotechnol. (2021) 39:64–73. doi: 10.1038/s41587-020-0613-1 32719479 PMC7854852

[56] Ajiro M Awaya T Kim YJ Iida K Denawa M Tanaka N. Therapeutic manipulation of IKBKAP mis-splicing with a small molecule to cure familial dysautonomia. Nat Commun. (2021) 12:4507. doi: 10.1038/s41467-021-24705-5 34301951 PMC8302731

[57] Matsushima S Ajiro M Iida K Chamoto K Honjo T Hagiwara M. Chemical induction of splice-neoantigens attenuates tumor growth in a preclinical model of colorectal cancer. Sci Transl Med. (2022) 14:eabn6056. doi: 10.1126/scitranslmed.abn6056 36449604

[58] Juneja VR McGuire KA Manguso RT LaFleur MW Collins N Haining WN. PD-L1 on tumor cells is sufficient for immune evasion in immunogenic tumors and inhibits CD8 T cell cytotoxicity. J Exp Med. (2017) 214:895–904. doi: 10.1084/jem.20160801 28302645 PMC5379970

[59] Lane RS Femel J Breazeale AP Loo CP Thibault G Kaempf A. IFNγ-activated dermal lymphatic vessels inhibit cytotoxic T cells in melanoma and inflamed skin. J Exp Med. (2018) 215:3057–74. doi: 10.1084/jem.20180654 PMC6279400 30381467

[60] Bajor M Graczyk-Jarzynka A Marhelava K Burdzinska A Muchowicz A Goral A. PD-L1 CAR effector cells induce self-amplifying cytotoxic effects against target cells. J Immunother Cancer. (2022) 10(12). doi: 10.1136/jitc-2021-002500 PMC8796262 35078921

[61] Nielsen C Ohm-Laursen L Barington T Husby S Lillevang ST. Alternative splice variants of the human PD-1 gene. Cell Immunol. (2005) 235:109–16. doi: 10.1016/j.cellimm.2005.07.007 16171790

[62] Wahid M Pratoomthai B Egbuniwe IU Evans HR Babaei-Jadidi R Amartey JO. Targeting alternative splicing as a new cancer immunotherapy-phosphorylation of serine arginine-rich splicing factor (SRSF1) by SR protein kinase 1 (SRPK1) regulates alternative splicing of PD1 to generate a soluble antagonistic isoform that prevents T cell exhaustion. Cancer Immunol Immunother. (2023) 72:4001–14. doi: 10.1007/s00262-023-03534-z PMC10700477 37973660

[63] Mahoney KM Shukla SA Patsoukis N Chaudhri A Browne EP Arazi A. A secreted PD-L1 splice variant that covalently dimerizes and mediates immunosuppression. Cancer Immunol Immunother. (2019) 68:421–32. doi: 10.1007/s00262-018-2282-1 PMC6426808 30564891

[64] Segal NH Parsons DW Peggs KS Velculescu V Kinzler KW Vogelstein B. Epitope landscape in breast and colorectal cancer. Cancer Res. (2008) 68:889–92. doi: 10.1158/0008-5472.Can-07-3095 18245491

[65] Peggs KS Segal NH Allison JP. Targeting immunosupportive cancer therapies: accentuate the positive, eliminate the negative. Cancer Cell. (2007) 12:192–9. doi: 10.1016/j.ccr.2007.08.023 17785201

[66] Westcott PMK Sacks NJ Schenkel JM Ely ZA Smith O Hauck H. Low neoantigen expression and poor T-cell priming underlie early immune escape in colorectal cancer. Nat Cancer. (2021) 2:1071–85. doi: 10.1038/s43018-021-00247-z PMC8562866 34738089

[67] Palmeri M Mehnert J Silk AW Jabbour SK Ganesan S Popli P. Real-world application of tumor mutational burden-high (TMB-high) and microsatellite instability (MSI) confirms their utility as immunotherapy biomarkers. ESMO Open. (2022) 7:100336. doi: 10.1016/j.esmoop.2021.100336 34953399 PMC8717431

[68] Ravaioli S Limarzi F Tumedei MM Palleschi M Maltoni R Bravaccini S. Are we ready to use TMB in breast cancer clinical practice? Cancer Immunol Immunother. (2020) 69:1943–5. doi: 10.1007/s00262-020-02682-w PMC11027700 32725361

[69] Samstein RM Lee CH Shoushtari AN Hellmann MD Shen R Janjigian YY. Tumor mutational load predicts survival after immunotherapy across multiple cancer types. Nat Genet. (2019) 51:202–6. doi: 10.1038/s41588-018-0312-8 PMC6365097 30643254

[70] Rizzo A Ricci AD. Biomarkers for breast cancer immunotherapy: PD-L1, TILs, and beyond. Expert Opin Investig Drugs. (2022) 31:549–55. doi: 10.1080/13543784.2022.2008354 34793275

[71] Zhu Y Zhu X Tang C Guan X Zhang W. Progress and challenges of immunotherapy in triple-negative breast cancer. Biochim Biophys Acta Rev Cancer. (2021) 1876:188593. doi: 10.1016/j.bbcan.2021.188593 34280474

[72] Zhao J Chen AX Gartrell RD Silverman AM Aparicio L Chu T. Immune and genomic correlates of response to anti-PD-1 immunotherapy in glioblastoma. Nat Med. (2019) 25:462–9. doi: 10.1038/s41591-019-0349-y PMC6810613 30742119

[73] Kembuan GJ Kim JY Maus MV Jan M. Targeting solid tumor antigens with chimeric receptors: cancer biology meets synthetic immunology. Trends Cancer. (2024) 10:312–31. doi: 10.1016/j.trecan.2024.01.003 PMC11006585 38355356

[74] Wang Z Cao YJ. Adoptive cell therapy targeting neoantigens: A frontier for cancer research. Front Immunol. (2020) 11:176. doi: 10.3389/fimmu.2020.00176 32194541 PMC7066210

[75] Biernacki MA Lok J Black RG Foster KA Cummings C Woodward KB. Discovery of U2AF1 neoantigens in myeloid neoplasms. J Immunother Cancer. (2023) 11(12). doi: 10.1136/jitc-2023-007490 PMC10729103 38164756

[76] Martín-Otal C Lasarte-Cia A Serrano D Casares N Conde E Navarro F. Targeting the extra domain A of fibronectin for cancer therapy with CAR-T cells. J Immunother Cancer. (2022) 10(8). doi: 10.1136/jitc-2021-004479 PMC9351345 35918123

[77] Huijbers EJ Ringvall M Femel J Kalamajski S Lukinius A Abrink M. Vaccination against the extra domain-B of fibronectin as a novel tumor therapy. FASEB J. (2010) 24:4535–44. doi: 10.1096/fj.10-163022 20634349

[78] Tang L Kong Y Wang H Zou P Sun T Liu Y. Demethylating therapy increases cytotoxicity of CD44v6 CAR-T cells against acute myeloid leukemia. Front Immunol. (2023) 14:1145441. doi: 10.3389/fimmu.2023.1145441 37180104 PMC10174291

[79] Tang L Huang H Tang Y Li Q Wang J Li D. CD44v6 chimeric antigen receptor T cell specificity towards AML with FLT3 or DNMT3A mutations. Clin Transl Med. (2022) 12:e1043. doi: 10.1002/ctm2.1043 36163632 PMC9513046

[80] Deng Z Zhan P Yang K Liu L Liu J Gao W. Identification of personalized neoantigen-based vaccines and immune subtype characteristic analysis of glioblastoma based on abnormal alternative splicing. Am J Cancer Res. (2022) 12:3581–600. PMC9442016 36119813

[81] Ito K Patel PN Gorham JM McDonough B DePalma SR Adler EE. Identification of pathogenic gene mutations in LMNA and MYBPC3 that alter RNA splicing. Proc Natl Acad Sci U.S.A. (2017) 114:7689–94. doi: 10.1073/pnas.1707741114 PMC5528995 28679633

[82] Feng L Guo M Jin C. Identification of alternative splicing and RNA-binding proteins involved in myocardial ischemia-reperfusion injury. Genome. (2023) 66:261–8. doi: 10.1139/gen-2022-0102 37466303

[83] Olivieri JE Dehghannasiri R Wang PL Jang S de Morree A Tan SY. RNA splicing programs define tissue compartments and cell types at single-cell resolution. Elife. (2021) 10. doi: 10.7554/eLife.70692 PMC8563012 34515025

[84] Wang L Liu Y Dai Y Tang X Yin T Wang C. Single-cell RNA-seq analysis reveals BHLHE40-driven pro-tumour neutrophils with hyperactivated glycolysis in pancreatic tumour microenvironment. Gut. (2023) 72:958–71. doi: 10.1136/gutjnl-2021-326070 PMC10086491 35688610

[85] Yu L Shen N Shi Y Shi X Fu X Li S. Characterization of cancer-related fibroblasts (CAF) in hepatocellular carcinoma and construction of CAF-based risk signature based on single-cell RNA-seq and bulk RNA-seq data. Front Immunol. (2022) 13:1009789. doi: 10.3389/fimmu.2022.1009789 36211448 PMC9537943

[86] Zhang X Zhu R Yu D Wang J Yan Y Xu K. Single-cell RNA sequencing to explore cancer-associated fibroblasts heterogeneity: “Single” vision for “heterogeneous” environment. Cell Prolif. (2024) 57:e13592. doi: 10.1111/cpr.13592 38158643 PMC11056715

[87] Zhang Z Pan Z Ying Y Xie Z Adhikari S Phillips J. Deep-learning augmented RNA-seq analysis of transcript splicing. Nat Methods. (2019) 16:307–10. doi: 10.1038/s41592-019-0351-9 PMC7605494 30923373

[88] Huang HH Ferguson ID Thornton AM Bastola P Lam C Lin YT. Proteasome inhibitor-induced modulation reveals the spliceosome as a specific therapeutic vulnerability in multiple myeloma. Nat Commun. (2020) 11:1931. doi: 10.1038/s41467-020-15521-4 32321912 PMC7176739

[89] Yang H Beutler B Zhang D. Emerging roles of spliceosome in cancer and immunity. Protein Cell. (2022) 13:559–79. doi: 10.1007/s13238-021-00856-5 PMC9232692 34196950

[90] Bao Y Zhang S Zhang X Pan Y Yan Y Wang N. RBM10 loss promotes EGFR-driven lung cancer and confers sensitivity to spliceosome inhibition. Cancer Res. (2023) 83:1490–502. doi: 10.1158/0008-5472.Can-22-1549 36853175

[91] Wang Z Wang S Qin J Zhang X Lu G Liu H. Splicing factor BUD31 promotes ovarian cancer progression through sustaining the expression of anti-apoptotic BCL2L12. Nat Commun. (2022) 13:6246. doi: 10.1038/s41467-022-34042-w 36271053 PMC9587234

[92] Bui TM Wiesolek HL Sumagin R. ICAM-1: A master regulator of cellular responses in inflammation, injury resolution, and tumorigenesis. J Leukoc Biol. (2020) 108:787–99. doi: 10.1002/jlb.2mr0220-549r PMC7977775 32182390

[93] Lin F Xu L Yuan R Han S Xie J Jiang K. Identification of inflammatory response and alternative splicing in acute kidney injury and experimental verification of the involvement of RNA−binding protein RBFOX1 in this disease. Int J Mol Med. (2022) 49(3). doi: 10.3892/ijmm.2022.5087 PMC8788925 35059728

[94] Rosenthal R Cadieux EL Salgado R Bakir MA Moore DA Hiley CT. Neoantigen-directed immune escape?in lung cancer evolution. Nature. (2019) 567:479–85. doi: 10.1038/s41586-019-1032-7 PMC6954100 30894752

[95] Cafri G Gartner JJ Zaks T Hopson K Levin N Paria BC. mRNA vaccine-induced neoantigen-specific T cell immunity in patients with gastrointestinal cancer. J Clin Invest. (2020) 130:5976–88. doi: 10.1172/jci134915 PMC7598064 33016924

[96] McGranahan N Furness AJ Rosenthal R Ramskov S Lyngaa R Saini SK. Clonal neoantigens elicit T cell immunoreactivity and sensitivity to immune checkpoint blockade. Science. (2016) 351:1463–9. doi: 10.1126/science.aaf1490 PMC4984254 26940869

